# *Nocardia* antimicrobial susceptibility testing by microdilution: evaluation of ready-to-use plate including tedizolid and meropenem

**DOI:** 10.1007/s10096-026-05588-0

**Published:** 2026-07-04

**Authors:** Mateo Dutarte, Charlotte Genestet, Gérard Lina, Oana Dumitrescu, Elisabeth Hodille

**Affiliations:** Laboratoire de biologie médicale de référence des nocardioses, Institut des agents infectieux, Laboratoire de biologie médicale multi-site, Groupement Hospitalier Nord, Hospices civils de Lyon, Hôpital de la Croix Rousse, 121 Boulevard des Canuts, 69004 Lyon, France

**Keywords:** *Nocardia*, Antimicrobial susceptibility testing, Microdilution, Meropenem, Tedizolid

## Abstract

**Purpose:**

Nocardiosis is a rare but serious infection occurring predominantly in immunocompromised patients. The CLSI microdilution antimicrobial susceptibility testing is recommended for all nocardioses. Ready-to-use plates are available (Sensititre™ RAPMYCOI), but antimicrobials of interest (e.g. tedizolid, meropenem) are lacking. At the request of the French nocardiosis reference medical biology laboratory, a plate including meropenem and tedizolid was produced (Sensititre™ FRNOCAR1).

**Methods:**

FRNOCAR1 was compared to RAPMYCOI using 133 clinical *Nocardia* isolates containing predominantly *N. farcinica* (23.3%), *N. cyriacigeorgica* (24.8%), and *N. nova* complex (21.1%). Acceptability was assessed using the FDA criteria (essential/category agreement; major, and very major error [VME] rates).

**Results:**

All criteria were respected for 6 antimicrobials; for 5, the upper limit of the VME rate exceeded the criteria, despite a point estimate of 0.0%, due to a lack of resistant strains in the collection. For linezolid, the VME rate could not be calculated due to the absence of linezolid-resistant strains. Tedizolid minimal inhibitory concentration (MIC)50/MIC90 were 2–3 dilutions lower than that of linezolid (0.5/0.5 µg/mL and 2/4 µg/mL, respectively). Imipenem was better than meropenem for *N. farcinica*; meropenem MIC was at least 2-dilutions lower than that of imipenem for *N. gamkensis* (8/8), *N. gipuzkoensis* (2/4), and *N. wallacei* (4/6).

**Conclusion:**

For the antimicrobials common to both plates, the FRNOCAR1 demonstrated good agreement compared with RAPMYCOI. FRNOCAR1 includes tedizolid and meropenem, for which MIC could be reported upon justified request, with a comment indicating that these results are pending further validation.

**Supplementary Information:**

The online version contains supplementary material available at https://doi.org/10.1007/s10096-026-05588-0.

## Introduction

Nocardiosis is a disease caused by ubiquitous environmental bacteria of the *Nocardia* genus; this rare but serious infection affects approximately 500 to 1000 individuals per year in the United States [1] and predominantly occurs in immunocompromised patients, such as those with solid organ and hematopoietic stem cell transplanted recipients, patients with auto-immune diseases treated by immunotherapies and/or corticosteroids, and patients with structural and functional lung impairments (cystic fibrosis, chronic obstructive pulmonary disease, and bronchiectasis) [1, 2]. Since high mortality rates due to disseminated nocardiosis have been reported, reaching up to 50% [3, 4], an early diagnosis and the initiation of appropriate targeted therapy are important. The cornerstone of antibiotic therapy for nocardiosis is trimethoprim/sulfamethoxazole (SXT), which is introduced empirically in combination with imipenem (IMI) or ceftriaxone (AXO), with or without amikacin (AMI) according to the severity and location of the infection [2, 5]. In case of intolerance or contraindication to the use of SXT, linezolid (LZD) is an alternative as it is always effective against all *Nocardia* species [2, 5]. Identification of the *Nocardia* species together with antimicrobial susceptibility testing (AST) is essential for patient management, as antimicrobial susceptibility profiles vary markedly among *Nocardia* species and treatment tolerance may differ between patients [1, 2]. Currently, the Clinical and Laboratory Standards Institute (CLSI) broth micro dilution method is the only recommended method to perform AST on *Nocardia* isolates (M24, 3rd edition; [6]). Ready-to-use plates with the label “in vitro diagnostic” (IVD) for aerobic Actinomycetes are available for microdilution susceptibility testing, such as the Sensititre^™^ Myco RAPMYCOI or NOCARDIA AST plates (Thermo Fischer Scientific, Waltham, MA, US). However, these IVD panels do not include certain antimicrobials that may have clinical utility such as tedizolid (TZD) and meropenem (MERO). TZD is reported to have good in vitro activity against *Nocardia* [7, 8] and there are several clinical case reports documenting successful use of TZD for treatment of disseminated nocardiosis [9–11], and pulmonary nocardiosis [12, 13]. Moreover, according to expert recommendations, nocardiosis antibiotic treatment should last for at least 3 months for cutaneous nocardiosis, 6 months for pulmonary nocardiosis, and 12 months for disseminated and central nervous system nocardiosis [2, 5]. Given the low risk of long-term adverse effects associated with TZD, notably for the risk of thrombocytopenia, neuropathy or lactic acidosis [14, 15], TZD may be an alternative option for patients unable to tolerate prolonged durations of linezolid (LZD) [16]. Concerning MERO, this is not typically recommended for the treatment of nocardiosis [2]. However, some in vitro data suggest that it may be effective against certain species with low minimal inhibitory concentration ([MIC], e.g. *N. abscessus*, which is usually resistant to imipenem [IMI], and *N. transvalensis* complex [17, 18]). Furthermore, several cases of *Nocardia* brain abscesses successfully treated with a combination of MERO plus trimethoprim/sulfamethoxazole (SXT), with or without neurosurgical intervention, have been reported [19–21]. Although it is not clear whether MERO susceptibility testing was performed in all cases, the use of MERO was justified in these case reports by a high central nervous system penetration [19]. It therefore seems beneficial for patient care to determine the MIC of these antimicrobials in the initial AST for *Nocardia*.

The manufacturer of Sensititre™ AST plates (Thermo Fischer Scientific) produced a new plate named FRNOCAR1 at the request of the French nocardiosis reference medical biology laboratory (*Laboratoire de biologie médicale de références des nocardioses*, LBMR des nocardioses) containing all the antimicrobials of interest for the treatment of nocardiosis (amikacin [AMI], amoxicillin-clavulanate [AUG2], cefotaxime [FOT], ceftriaxone [AXO], ciprofloxacin [CIP], clarithromycin [CLA], doxycycline [DOX], IMI, LZD, MERO, minocycline [MIN], moxifloxacin [MXF], TZD, tigecycline [TGC], tobramycin [TOB], SXT), with a range of concentrations for each antibiotic adapted to the distribution of MIC in *Nocardia*. As this plate does not have IVD marking, the main aim of the present study was to compare the MIC results obtained with the FRNOCAR1 with those obtained using the reference IVD RAPMYCOI plate. Ensuring consistency between the two plates before routine implementation of the FRNOCAR1 by the LBMR des nocardioses was an integral part of the accreditation process in accordance with ISO 15,189. The secondary objective was to generate MIC data for TZD and MERO using a large collection of clinical *Nocardia* isolates, through the FRNOCAR1.

## Materials and methods

### Nocardia isolates

A collection of 133 *Nocardia* clinical isolates isolated between 2012 and 2015 from different geographical locations (Provincial Laboratory for Public Health, Edmonton, AB, Canada; the Karolinska University Hospital, Stockholm, Sweden; and the Infection Control Lab [NHLS] and Department of Clinical Microbiology and Infectious Diseases, Charlotte Maxeke Hospital, Johannesburg, South Africa), collected by the EUropean Committee on Antimicrobial Susceptibility Testing (EUCAST), were used in the present study. *Nocardia* isolates were cultured on Columbia agar with 5% sheep blood (COS; bioMérieux, Marcy l’Etoile, France) for 48 to 72 h, in an aerobic atmosphere. Then, isolated colonies were used to perform *Nocardia* identification by 16 S rRNA/hsp65 amplification and sequencing, the gold standard for *Nocardia* species identification, as previously described [22].

### Microdilution AST

Microdilution AST was performed using the RAPMYCOI (Thermo Fischer Scientific; Figure S1) according the manufacturer’s instruction, as well as the new plate FRNOCAR1 (Thermo Fischer Scientific; Figure S1) with the same inoculum. Briefly, *Nocardia* colonies cultured on COS agar for 48 to 72 h were emulsified using a moistened swab in demineralized water to obtain a bacterial suspension at 0.5 McFarland turbidity standard. Then 50 µL of the suspension were transferred into cation-adjusted Mueller-Hinton broth (Thermo Fischer Scientific). RAPMYCOI and FRNOCAR1 were inoculated with 100 µL of suspension added to each well. RAPMYCOI and FRNOCAR1 were then incubated at 35 °C ± 2 °C for 48 h or 72 h, according to the growth of the positive control. RAPMYCOI and FRNOCAR1 were read using the Sensititre™ manual viewer and MIC were collected. Interpretative categories, Susceptible (S), Intermediate (I), and Resistant (R), were determined using the CLSI critical concentrations for *Nocardia* spp and other aerobic actinomycetes [23], expect for cefepime (FEP), FOT, cefoxitin (FOX), MERO, and TIG, for which interpretative categories were determined using the pharmacokinetics/pharmacodynamics (PK/PD) breakpoint of *Comité de l’Antibiogramme de la Société Française de Microbiologie* (CA-SFM) 2025 [24]. For these antimicrobials, the clinical category “I” means susceptible, increased exposure. *Staphylococcus aureus* ATCC 29,213 was used as a quality control to check the batches of RAPMYCOI and FRNOCAR1 used. Reproducibility tests for the FRNOCAR1 were carried out in the same laboratory using two strains: *S. aureus* ATCC 29,213 with 5 replicates and *N. asteroides* ATCC19247 with 20 replicates.

### Analysis

The MIC50 and MIC90 represented the concentrations inhibiting 50% and 90% of the tested isolates, respectively. For the 13 antimicrobials contained in both plates (AMI, AUG2, AXO, CLA, CIP, DOX, IMI, LZD, MIN, MXF, SXT, TGC, and TOB), the US Food and Drug Administration (FDA) criteria were used to assess the acceptability of FRNOCAR1 for each antibiotic [25]: percent category agreement (CA) and essential agreement (EA) > 89.9%, a major error (ME: the reference category result is S and the new device result is R) rate ≤ 3% based on the number of susceptible organisms tested, a very major error (VME: the reference category result is R and the new device result is S) rate based on the number of resistant organisms tested for which the upper limit of the 95% confidence interval (95% CI) is < 7.5% and the lower limit of the 95% CI is < 1.5%. A minor error (mE) is when the reference category is R or S and the new device reported category is I, or when the reference category is I and the new device reported category is S or R, based on the total number of organisms tested.

## Results

### Nocardia isolates diversity

The collection contained 133 *Nocardia* strains from 18 different species (Table 1), with a predominant (69.2%) number of strains of the species most frequently found in human pathology (*N. cyriacigeorgica*, *N. farcinica*, and *N. nova* complex [26–29].

**Table 1:** Species of 133 *Nocardia* isolates included in the study

Nocardia species	Number (%) *N* = 133
*N. cyriacigeorgica*	33 (24.8)
*N. farcinica*	31 (23.3)
*N. nova complex*	28 (21.1)
*N. nova*	25
*N. veterana*	2
*N. elegans*	1
*N. gamkensis*	10 (7.5)
*N. brasiliensis*	8 (6.0)
*N. wallacei*	6 (4.5)
*N. asiatica*	4 (3.0)
*N. gipuzkoensis*	4 (3.0)
*N. puris*	2 (1.5)
*N. barduliensis*	1 (0.75)
*N. flavorosea*	1 (0.75)
*N. otitidiscavarium*	1 (0.75)
*N. pseudobrasiliensis*	1 (0.75)
*N. shimofusensis*	1 (0.75)
*N. terpenica*	1 (0.75)
*N. thailandica*	1 (0.75)

### Antibiotic susceptibility profiles

**Fig. 1:**
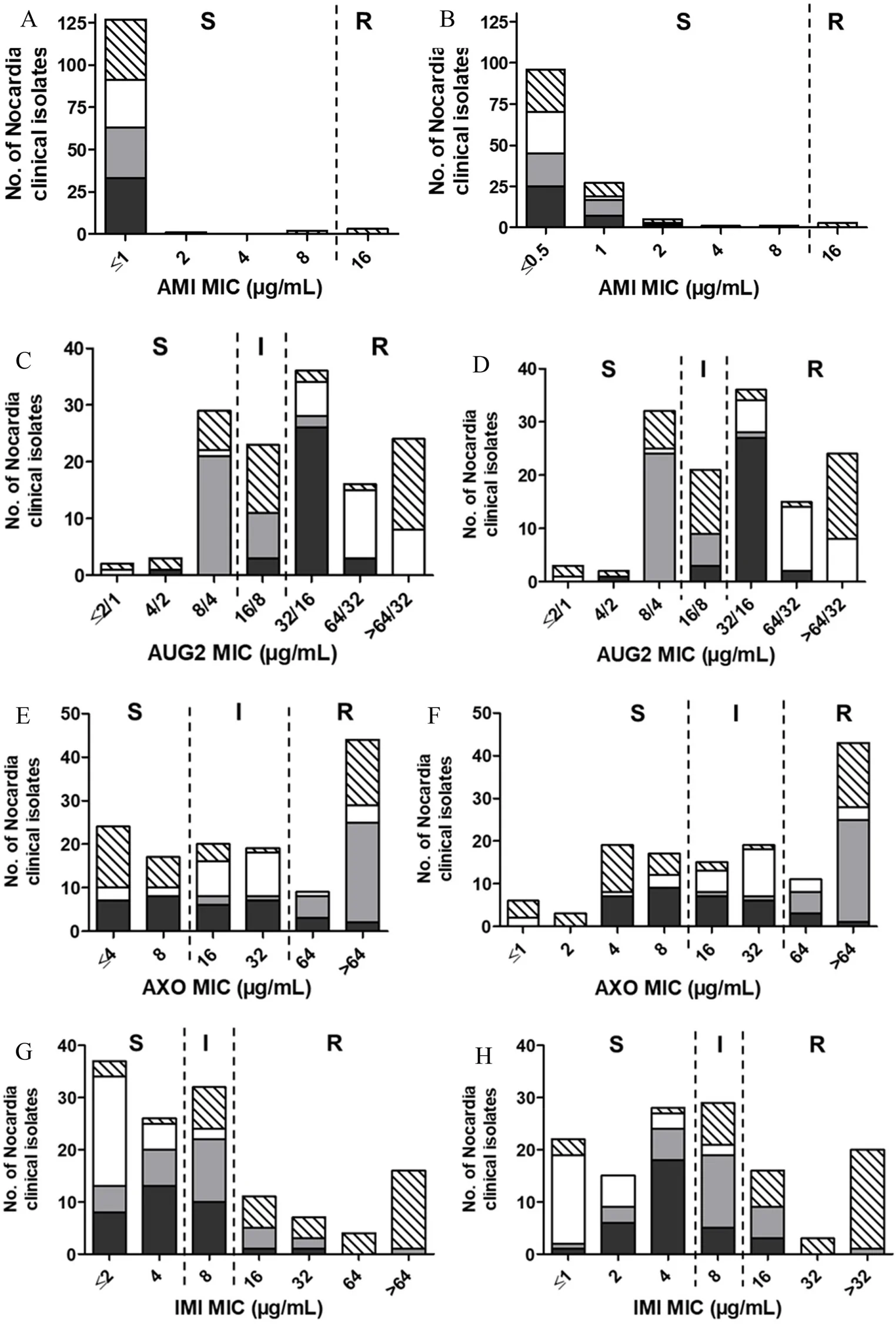

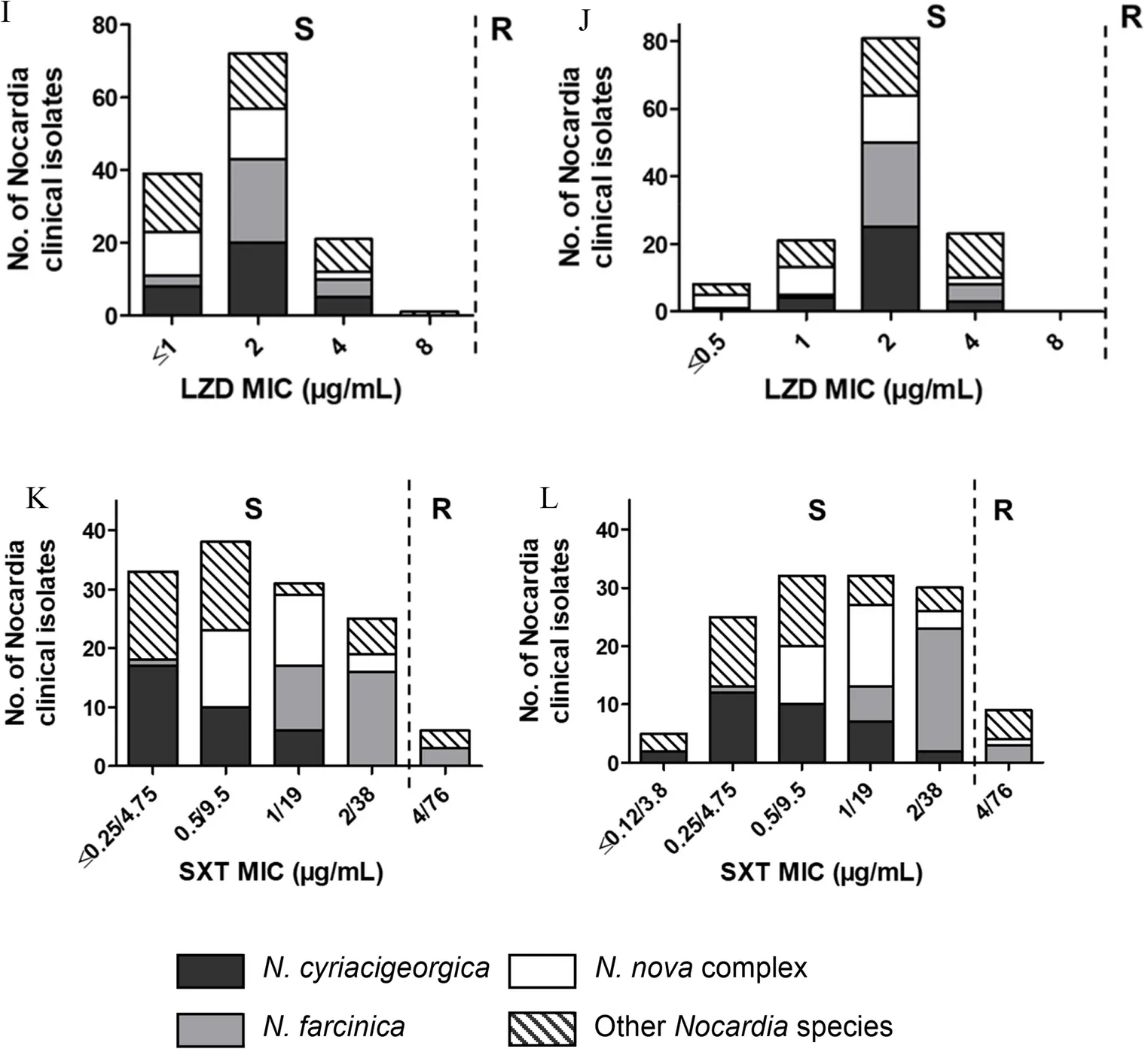
*Nocardia* (n=133) MIC distribution of amikacin (AMI, panels **A** and **B**), amoxicillin-clavulanate (AUG2, panels **C** and **D**), ceftriaxone (AXO, panels **E** and **F**), imipenem (IMI, panels **G** and **H**), linezolid (LZD, panels **I** and **J**), and trimethoprim-sulfamethoxazole (SXT, panels **K** and **L**), according to species. MIC derived from data originating from RAPMYCOI plate for the panels **A**, **C**, **E**, **G**, **I** and **K**, and from FRNOCAR1 plate for the panels **B**, **D**, **F**, **H**, **J** and **L**. S: Susceptible; I: Intermediate; R: Resistant. Dotted lines: critical concentrations of CLSI for Nocardia spp and other aerobic actinomycetes for all antimicrobials

Using RAPMYCOI, susceptibility rates among *Nocardia* strains were 100.0% for LZD, 97.7% for AMI, and 95.5% for SXT (Fig. 1; Table 2). Comparable results were obtained with FRNOCAR1, with susceptibility rates of 100.0%, 97.7%, and 93.2%, respectively (Fig. 1; Table 2). Using RAPMYCOI, only 3 strains were AMI-R (3 *N. wallacei*) and 6 strains were SXT-R (1 *N. otitidiscavrium*, 1 *N. wallacei*, 1 *N. thailandica*, and 3 *N. farcinica*; Fig. 1). FRNOCAR1 identified the same AMI-R and SXT-R strains, but additionally classified 3 more isolates as SXT-R, namely 1 *N. pseudobrasiliensis*, 1 *N. veterana*, and 1 *N. puris* (Fig. 1).

**Table 2:** Antimicrobial susceptibility profiles and MIC (µg/mL) values to 18 antibiotics of 133 *Nocardia* isolates included in the study using RAPMYCOI and FRNOCAR1 plates

Antibiotic	Breakpoint		*N. cyriacigeorgica* (*n* = 33)		*N. farcinica* (*n* = 31)		*N. nova* complex (*n* = 28)		All *Nocardia* (*n* = 133)	
			RAPMYCOI	FRNOCAR1	RAPMYCOI	FRNOCAR1	RAPMYCOI	FRNOCAR1	RAPMYCOI	FRNOCAR1
AMI	S ≤ 8, *R* ≥ 16	MIC50	≤ 1	≤ 0.5	≤ 1	≤ 0.5	≤ 1	≤ 0.5	≤ 1	≤ 0.5
		MIC90	≤ 1	1	≤ 1	1	≤ 1	1	≤ 1	1
		Range	≤ 1	≤ 0.5–2	≤ 1–2	≤ 0.5–2	≤ 1	≤ 0.5–2	≤ 1–16	≤ 0.5–16
		S/R (%)	100.0/0.0	100.0/0.0	100.0/0.0	100.0/0.0	100.0/0.0	100.0/0.0	97.7/2.3	97.7/2.3
AUG2	S ≤ 8/4, *R* ≥ 32/16	MIC50	32/16	32/16	8/4	8/4	64/32	64/32	32/16	32/16
		MIC90	32/16	32/16	16/8	16/8	> 64/32	> 64/32	> 64/32	> 64/32
		Range	4/2–64/32	4/2–64/32	8/4–32/16	8/4–32/16	≤ 2/1 - >64/32	≤ 2/1 - >64/32	≤ 2/1 - >64/32	≤ 2/1 - >64/32
		S/I/R (%)	3.0/9.1/87.9	3.0/9.1/87.9	67.7/25.8/6.5	77.4/19.4/3.2	7.1/21.4/71.5	7.1/0.0/92.9	25.6/17.3/57.1	27.8/15.8/56.4
AXO	S ≤ 8, *R* ≥ 64	MIC50	16	16	> 64	> 64	32	32	32	32
		MIC90	64	64	> 64	> 64	> 64	> 64	> 64	> 64
		Range	≤ 4 - > 64	4 - > 64	16 - >64	16 - >64	≤ 4 - >64	≤ 1 - >64	≤ 4 - >64	≤ 1 - >64
		S/I/R (%)	45.4/39.4/15.2	48.5/39.4/12.1	0.0/9.7/90.3	0.0/6.5/93.5	17.9/64.2/17.9	21.4/57.2/21.4	30.8/29.3/39.9	33.8/25.6/40.6
CIP	S ≤ 1, *R* ≥ 4	MIC50	> 4	> 8	2	2	> 4	> 8	> 4	8
		MIC90	> 4	> 8	> 4	4	> 4	> 8	> 4	> 8
		Range	2 - >4	4 - >8	≤ 0.12 - >4	≤ 0.25–8	> 4	8 - >8	≤ 0.12 - >4	≤ 0.5 - >8
		S/I/R (%)	0/3.0/97.0	0.0/0.0/100.0	45.2/19.3/35.5	41.9/25.8/32.3	0.0/0.0/100.0	0.0/0.0/100.0	16.5/6.0/77.5	15.8/6.8/77.4
CLA	S ≤ 2, *R* ≥ 8	MIC50	> 16	> 16	> 16	> 16	≤ 0.06	≤ 1	> 16	> 16
		MIC90	> 16	> 16	> 16	> 16	≤ 0.06	≤ 1	> 16	> 16
		Range	4 - >16	2 - >16	8 - >16	8 - >16	≤ 0.06–4	≤ 1–4	≤ 0.06 - >16	≤ 1 - >16
		S/I/R (%)	0.0/15.2/84.8	3.0/9.1/87.9	0.0/0.0/100.0	0.0/0.0/100.0	96.4/3.6/0.0	96.4/3.6/0.0	25.6/9.7/64.7	26.3/8.3/65.4
DOX	S ≤ 1, *R* ≥ 8	MIC50	2	2	4	4	16	8	4	4
		MIC90	4	4	8	8	> 16	16	16	16
		Range	≤ 0.12–8	≤ 0.5–4	1–16	1–16	1 - >16	1 - >16	≤ 0.12 - >16	≤ 0.5 - >16
		S/I/R (%)	24.2/72.8/3.0	24.2/75.8/0.0	3.2/58.1/38.7	3.2/67.8/29.0	3.6/7.1/89.3	3.6/14.3/82.1	18.0/48.9/33.1	16.5/54.9/28.6
FEP	S ≤ 4, *R* ≥ 16*	MIC50	32	/	> 32	/	16	/	> 32	/
		MIC90	> 32	/	> 32	/	> 32	/	> 32	/
		Range	8 - >32	/	32 - >32	/	1 - >32	/	1 - >32	/
		S/I/R (%)	0/3.1/96.9	/	0/0/100.0	/	14.3/10.7/75.0	/	5.3/8.2/86.5	/
FOT	S ≤ 1, *R* ≥ 4*	MIC50	/	8	/	> 64	/	32	/	32
		MIC90	/	64	/	> 64	/	> 64	/	> 64
		Range	/	4 - >64	/	16 - >64	/	≤ 1 - >64	/	≤ 1 - >64
		S/I/R (%)	/	0/0/100.0	/	0/0/100.0	/	7.1/92.9	/	6.8/93.2
FOX	S ≤ 4, *R* > 4	MIC50	> 128	/	> 128	/	128	/	> 128	/
		MIC90	> 128	/	> 128	/	> 128	/	> 128	/
		Range	64–128	/	64–128	/	4 - >128	/	4 - >128	/
		S/R (%)	0.0/100.0	/	0.0/100.0	/	3.6/96.4	/	0.8/99.2	/
IMI	S ≤ 4, *R* ≥ 16	MIC50	4	4	8	8	≤ 2	≤ 1	8	8
		MIC90	8	8	16	16	4	4	> 64	> 32
		Range	≤ 2–32	≤ 1–16	≤ 2–32	≤ 1–32	≤ 2–8	≤ 1–8	≤ 2 - >64	≤ 1 - >32
		S/I/R (%)	63.6/30.3/6.1	75.7/15.2/9.1	38.7/38.7/22.6	32.2/45.2/22.6	92.9/7.1/0.0	92.9/7.1/0.0	47.4/24.1/28.5	48.9/21.8/29.3
LZD	S ≤ 8	MIC50	2	2	2	2	2	2	2	2
		MIC90	4	2	4	4	2	2	4	4
		Range	≤ 1–4	≤ 0.5–4	≤ 1–4	1–4	≤ 1–4	≤ 0.5–4	≤ 1–8	≤ 0.5–4
		S/R (%)	100.0/0.0	100.0/0.0	100.0/0.0	100.0/0.0	100.0/0.0	100.0/0.0	100.0/0.0	100.0/0.0
MERO	S ≤ 2, *R* ≥ 16*	MIC50	/	8	/	16	/	1	/	8
		MIC90	/	16	/	16	/	4	/	16
		Range	/	4–16	/	8–32	/	1–4	/	1 - >32
		S/I/R (%)	/	0.0/81.8/18.2	/	0.0/22.6/77.4	/	75.0/25.0/0.0	/	18.8/46.6/34.6
MIN	S ≤ 1, *R* ≥ 8	MIC50	2	2	4	4	4	4	4	4
		MIC90	4	4	4	4	4	4	4	4
		Range	≤ 1–4	≤ 0.5–4	≤ 1–4	1–8	2–8	2–8	≤ 1–8	≤ 0.5–8
		S/I/R (%)	12.1/87.9/0.0	12.1/87.9/0.0	3.2/96.8/0.0	3.2/93.6/3.2	0.0/96.4/3.6	0.0/96.4/3.6	14.3/82.7/3.0	13.5/80.5/6.0
MXF	S ≤ 1, *R* ≥ 4	MIC50	8	8	1	1	8	8	4	4
		MIC90	> 8	> 8	2	2	> 8	> 8	> 8	> 8
		Range	1 - >8	1 - >8	≤ 0.25–8	≤ 0.5–4	2 - >8	2 - >8	≤ 0.25 - >8	≤ 0.5 - >8
		S/I/R (%)	3.0/3.0/94.0	3.0/6.1/90.9	64.5/25.8/9.7	61.3/29.0/9.7	0.0/7.1/92.9	0.0/3.6/96.4	23.3/14.3/62.4	23.3/15.0/61.7
SXT	S ≤ 2/38, *R* ≥ 4/76	MIC50	≤ 0.25/4.75	0.5/9.5	2/38	2/38	1/19	1/19	0.5/9.5	0.5/9.5
		MIC90	1/19	1/19	2/38	2/38	2/38	2/38	2/38	2/38
		Range	≤ 0.25/4.75–1/19	≤ 0.12/2.38–2/38	≤ 0.25/4.75–4/76	0.25/4.75–4/76	0.5/9.5–2/38	0.5/9.5–4/76	≤ 0.25/4.75–4/76	≤ 0.12/2.38–4/76
		S/R (%)	100.0/0.0	100.0/0.0	90.3/9.7	90.3/9.7	100.0/0.0	96.4/3.6	95.5/4.5	93.2/6.8
TGC	S ≤ 0.5, *R* > 0.5*	MIC50	0.5	0.5	1	2	1	1	1	1
		MIC90	1	1	2	2	4	4	2	2
		Range	0.25–1	0.25–1	0.25–4	0.25–4	0.5 - >4	0.5 - >4	0.25 - >4	0.25 - >4
		S/R (%)	75.8/24.2	84.8/15.2	12.9/87.1	6.5/93.5	21.4/78.6	17.9/82.1	46.6/53.4	42.9/57.1
TOB	S ≤ 4, *R* ≥ 16	MIC50	≤ 1	≤ 1	16	16	> 16	> 16	1	2
		MIC90	≤ 1	≤ 1	> 16	> 16	> 16	> 16	> 16	> 16
		Range	≤ 1	≤ 1	8 - >16	8 - >16	≤ 1 - >16	4 - >16	≤ 1 - >16	≤ 1 - >16
		S/I/R (%)	100.0/0.0/0.0	100.0/0.0/0.0	0.0/6.5/93.5	0.0/6.5/93.5	3.6/7.1/89.3	3.6/7.1/89.3	51.1/3.0/45.9	51.1/3.0/45.9
TZD	/	MIC50	/	0.5	/	0.5	/	0.25	/	0.5
		MIC90	/	0.5	/	0.5	/	0.5	/	0.5
		Range	/	0.25–1	/	0.25–1	/	≤ 0.06–0.5	/	≤ 0.06–2
		S/I/R (%)	/	N. D	/	N. D	/	N. D	/	N. D

*AMI* amikacin, *AUG2* amoxicillin-clavulanate, *AXO* ceftriaxone, *CIP* ciprofloxacin, *CLA* clarithromycin, *DOX* doxycycline, *FEP* cefepime, *FOT* cefotaxime, *FOX* cefoxitine, *IMI* imipenem, *LZD* linezolid, *MERO* meropenem, *MIN* minocycline, *MXF* moxifloxacin, *SXT* trimethoprim-sulfamethoxazole, *TGC* tigecycline, *TOB* tobramycin, *TZD* tedizolid

*MIC50* minimal inhibitory concentration 50, *MIC90* minimal inhibitory concentration 90

Interpretative categories S (Susceptible), I (Intermediate), and R (Resistant) determined using the CLSI critical concentrations for *Nocardia* spp and other aerobic actinomycetes, expect for * determined using the PK/PD breakpoint of *Comité de l’Antibiogramme de la Société Française de Microbiologie* (CA-SFM) 2025 [24]. For these antimicrobials, I = Susceptible, increased exposure

*N.D* not determined;/: value not available

Regarding beta-lactams, the susceptibility to antibiotics depended heavily on the species and corresponded to the resistance profile expected [23] for a given species; with RAPMYCOI and FRNOCAR1, 97.0% of *N. cyriacigerogica* were non-S (R or I) to AUG2, as were 92.9% of *N. nova*, and 100.0% of *N. farcinica* were non-S to AXO (Table 2; Fig. 1).

**Fig. 2:**
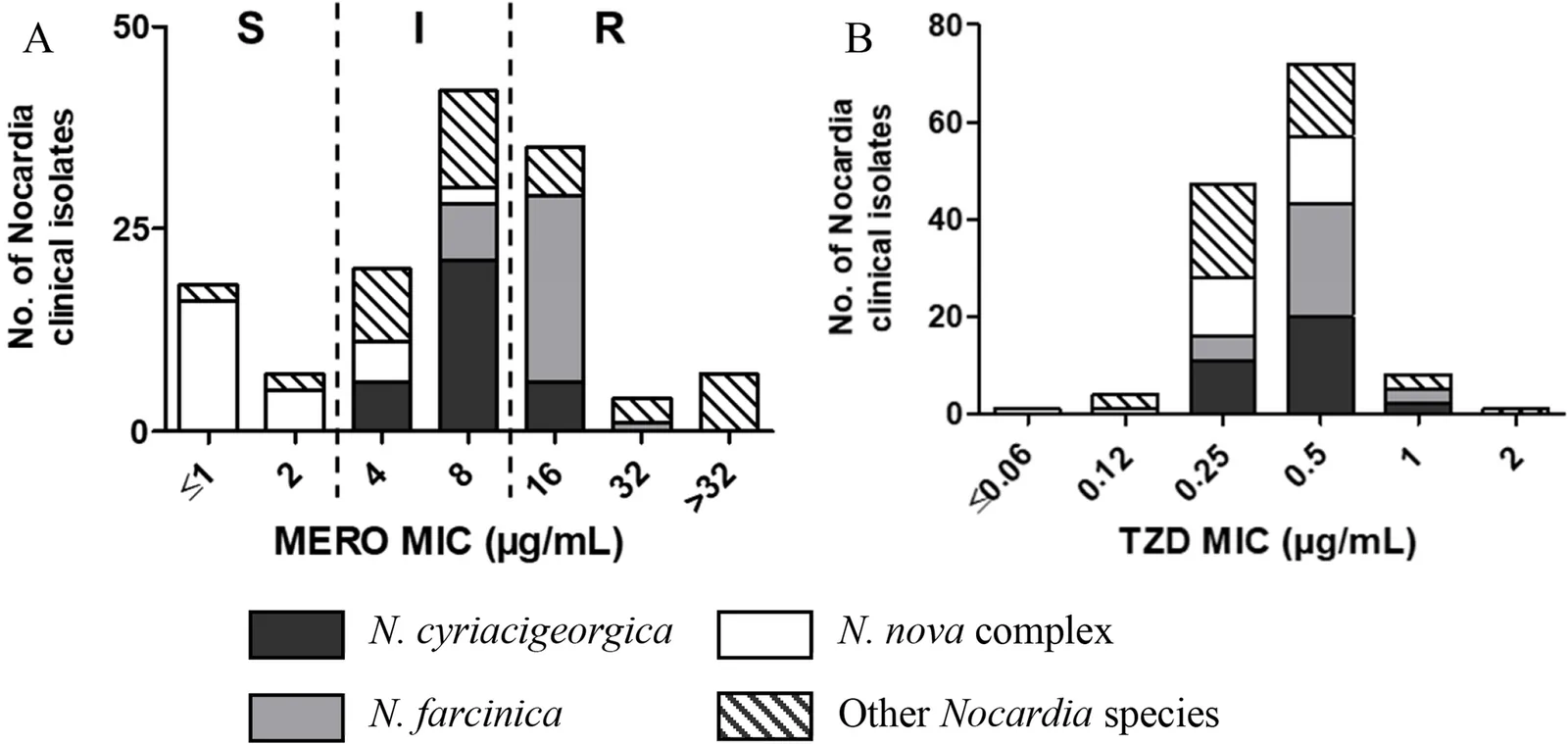
*Nocardia* (n=133) MIC distribution of meropenem (MERO, panel **A**) and tedizolid (TZD, panel **B**) according to species. MIC derived from data originating from FRNOCAR1 plate for the panels **A** and **B**. S: Susceptible; I: Intermediate; R: Resistant. Dotted lines: PK/PD breakpoint of *Comité de l’Antibiogramme de la Société Française de Microbiologie* (CA-SFM) 2025 for MERO

Regarding carbapenems, the IMI MIC50 were 1 dilution lower than that of MERO for *N. cyriacigeorgica* (4 µg/mL and 8 µg/mL, respectively) and *N. farcinica* (8 µg/mL and 16 µg/mL, respectively). For *N. nova* complex, the MERO MIC were the same as those for IMI (Table 2; Figs. 1 and 2).

**Fig. 3:**
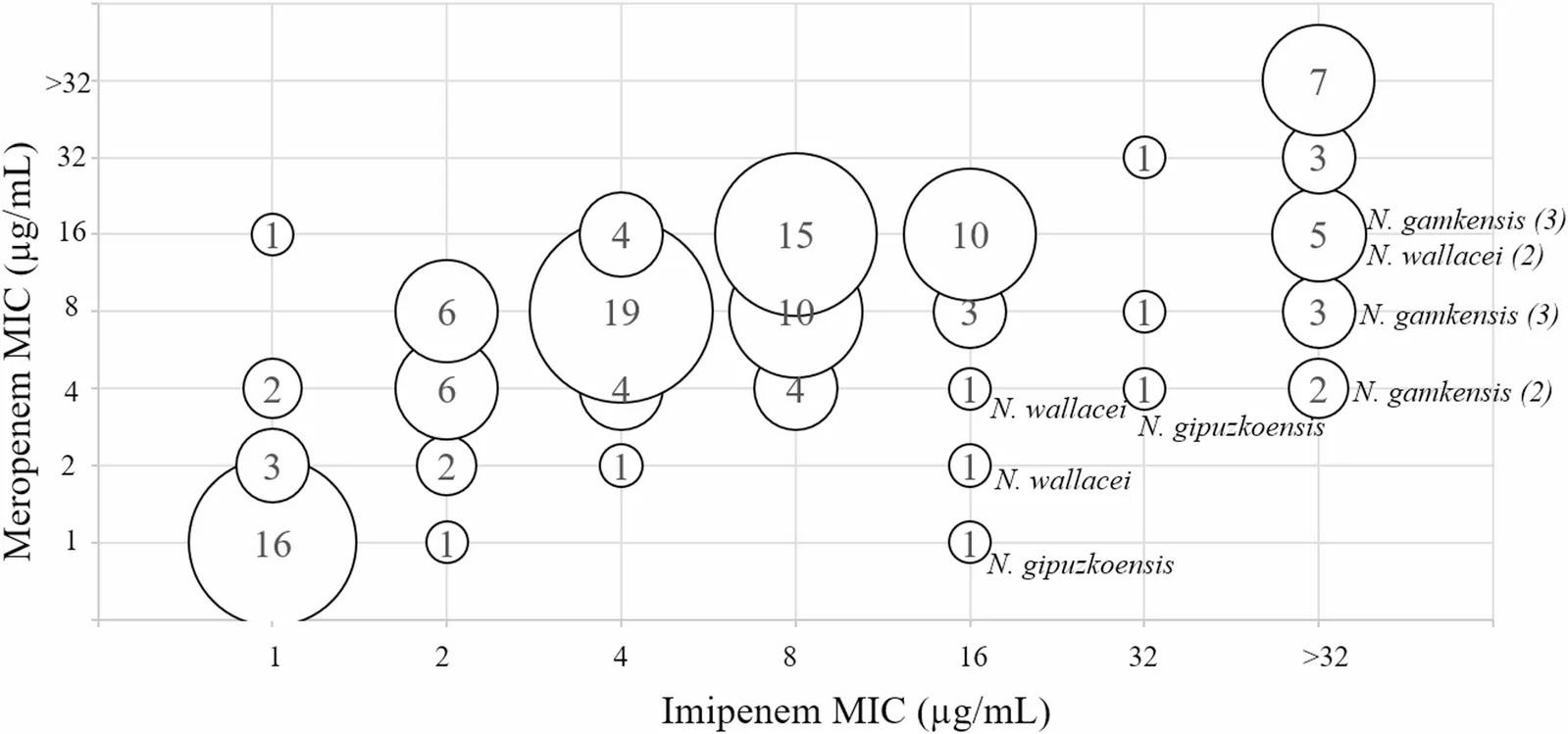
*Nocardia* strains (n = 133) distribution according to MIC imipenem (on the x-axis) and MIC meropenem (on the y-axis). The surface area of the bubbles is proportional to the number of individuals. MIC data derived from data originating from FRNOCAR1 plate

Moreover, among the IMI-R strains (MIC ≥ 16 µg/mL), certain strains displayed a MERO MIC at least 2 dilutions lower and these belonged mainly to the species *N. gamkensis* (8/8), *N. gipuzkoensis* (2/4), and *N. wallacei* (4/6; Fig. 3).

Regarding oxazolidinones (Figs. 1 and 2), the TZD MIC50 was 2 dilutions lower than that of LZD (0.5 µg/mL and 2 µg/mL respectively) and TZD MIC90 was 3 dilutions lower than that of LZD (0.5 µg/mL and 4 µg/mL, respectively; Table 2). Only one of the 133 strains tested had a high MIC for TZD (2 µg/mL). This was a strain of *N. brasiliensis* that also had a high MIC for LZD (8 µg/mL with RAPMYCOI and 4 µg/mL with FRNOCAR1 Fig. 1).

### Comparison between RAPMYCOI and FRNOCAR1

All FDA criteria were respected for AUG2, AXO (Fig. 1), CIP, CLA, MXF, and TOB (Figure S2, Table 3). For, IMI (Fig. 1) and DOX (Figure S2), the CA did not meet the FDA criteria, but the EA was very good and there only mE (Table 3). For AMI (Fig. 1), DOX (Figure S2), IMI, MIN (Figure S2) and SXT (Fig. 1), the upper limit of the VME rate exceeded the FDA criteria, despite a point estimate and a lower limit of the 95% CI of 0.0% (Table 3). For LZD (Fig. 1), the VME rate could not be calculated due to the absence of LZD-resistant strains and for TGC (Figure S2), despite an EA of 100.0%, the other parameters did not meet the FDA criteria (Table 3).

**Table 3:** Essential agreement (EA), category agreement (CA), minor error (mE), major error (ME), and very major error (VME) rates between Sensititre™ Myco RAPMYCOI (reference) and FRNOCAR1 (new device) AST plates for 13 antimicrobials

	EA (MIC = MIC* +/- 1 dilution)		CA S*/S; I*/I; R*/*R*		mE S* or R*/I; I*/S or *R*		ME S*/*R*		VME R*/S		Total isolates R* (*n*)	Total isolates S* (*n*)	Total isolates I* (*n*)
	*n*	% [95% CI] (/total isolates)	*n*	% [95% CI] (/total isolates)	*n*	% [95% CI] (/total isolates)	*n*	% [95% CI] (/isolates S*)	*n*	% [95% CI] (/isolates R*)			
AMI	133	**100.0** [97.3; 100.0]	133	**100.0** [97.3; 100.0]	0	N.D.	0	**0.0** [0.0; 2.8]	0	0.0 **[0.0**; 70.8]	3	130	0
AUG2	133	**100.0** [97.3; 100.0]	127	**95.5** [90.4; 98.3]	6	4.5 [1.7; 9.6]	0	**0.0** [0.0; 10.3]	0	0.0 **[0.0; 4.7]**	76	34	23
AXO	127	**95.5** [90.4; 98.3]	120	**90.2** [83.7; 94.7]	13	9.8 [5.3; 16.1]	0	**0.0** [0.0; 8.6]	0	0.0 **[0.0; 6.7]**	53	41	39
CIP	130	**97.7** [93.5; 99.5]	130	**97.7** [93.5; 99.5]	3	2.3 [0.5; 6.5]	0	**0.0** [0.0; 15.4]	0	0.0 **[0.0; 3.5]**	103	22	8
CLA	133	**100.0** [97.3; 100.0]	131	**98.5** [94.7; 99.8]	2	1.5 [0.2; 5.3]	0	**0.0** [0.0; 10.3]	0	0.0 **[0.0; 4.2]**	86	34	13
DOX	133	**100.0** [97.3; 100.0]	115	86.5 [79.5; 91.8]	18	13.5 [8.2; 20.5]	0	**0.0** [0.0; 14.2]	0	0.0 **[0.0**; 8.0]	44	24	65
IMI	131	**98.5** [94.7; 99.8]	118	88.7 [82.1; 93.5]	15	11.3 [6.5; 17.9]	0	**0.0 ** [0.0; 5.7]	0	0.0 **[0.0**; 9.3]	38	63	32
LZD	133	**100.0** [97.3; 100.0]	133	**100.0** [97.3; 100.0]	0	N.D.	0	**0.0** [0.0; 2.7]	0	N.D.	0	133	0
MIN	133	**100.0** [97.3; 100.0]	122	**91.7** [85.7; 95.8]	11	8.3 [4.2; 14.3]	0	**0.0** [0.0; 17.6]	0	**0.0 ** **[0.0;** 60.2]	4	19	110
MXF	133	**100.0** [97.3; 100.0]	126	**94.7** [89.4; 97.9]	7	5.3 [2.1; 10.5]	0	**0.0** [0.0; 11.2]	0	0.0 **[0.0; 4.3]**	83	31	19
SXT	131	**98.5** [94.7; 99.8]	130	**97.7** [93.5; 99.5]	0	N.D.	3	**2.4** [0.5; 6.9]	0	0.0 **[0.0;** 45.9]	6	127	0
TGC	133	**100.0** [97.3; 100.0]	117	87.9 [81.2; 92.9]	0	N.D.	10	16.1 [8.0; 27.7]	6	8.5 [3.2; 17.5]	71	62	0
TOB	133	**100.0** [97.3; 100.0]	131	**98.5** [94.7; 99.8]	2	1.5 [0.2; 5.3]	0	**0.0** [0.0; 5.3]	0	0.0 **[0.0; 5.9]**	61	68	4

*95% CI* confidence interval 95%, *MIC* minimal inhibitory concentration determined using Sensititre™ FRNOCAR1, *S, R, I* clinical category determined using Sensititre™ FRNOCAR1, MIC* minimal inhibitory concentration determined using Sensititre™ Myco RAPMYCOI, S*, R*, I* clinical category determined using Sensititre™ Myco RAPMYCOI, *N.D.* not determined, *AMI* amikacin, *AXO* ceftriaxone, *AUG2* amoxicillin-clavulanate, *CIP* ciprofloxacin, *CLA* clarithromycin, *DOX* doxycycline, *IMI* imipenem, *LZD* linezolid, *MIN* minocycline, *MXF* moxifloxacin, *TGC* tigecycline, *TOB* tobramycin, *SXT* trimethoprim-sulfamethoxazole. In bold: conditions that reached the FDA criteria (EA and CA > 90%, ME rate ≤ 3%, VME rate 95% CI [≤ 1.5%; ≤ 7.5%])

### Reproducibility assay

The best reproducibility, assuming the off-scale result within one well from the mode, was 100.0% for all antibiotics tested with the FRNOCAR1 (Table S1-S15). The worst reproducibility, assuming the off-scale result is greater than one well from the mode, was 100.0% for all antibiotics tested with the FRNOCAR1, except for AXO, for which this was 92.0% (Table S1-S15). In addition, the MIC found with the FRNOCAR1 for *S. aureus* ATCC29213 strain was within the expected range of CA-SFM 2025 100.0% of the time for AMI, CLA, CIP, DOX, LZD, MIN, SXT, TOB, and TZD. For AUG2 and MXF, the FRNOCAR1 did not allow sufficiently low MIC values to be obtained to fall within the expected range (Tables S1-S15). For AXO, FOT, IMI and MERO for *S. aureus* ATCC 29,213, and for *N. asteroides* ATCC19247, there were no expected MIC values in the recommendations of learned societies (CLSI or CA-SFM).

## Discussion

With regard to antibiotic susceptibility profiles, the present study shows that the number of antimicrobials that can be used in probabilistic antibiotic therapy with guaranteed effectiveness in cases of nocardiosis is low (LZD, AMI, SXT), that no beta-lactams have a high susceptibility rate (> 90%) against *Nocardia*, and that species identification is essential for selecting the best antimicrobial among beta-lactams. It is of note that FOX and FEP were clearly unsuitable for the treatment of nocardiosis (0.8% and 5.3% of susceptible isolates, and Figure S3), and therefore these 2 antimicrobials can be omitted from the plate for *Nocardia* AST. For other cephalosporins, MIC50 for AXO and FOT were close, as were the MIC90, for all categories of *Nocardia* tested (*N. cyriacigeorgica*, *N. farcinica*, *N. nova* complex and all *Nocardia*). However, the frequency of FOT-S isolates was drastically lower than that of AXO-S (Figure S4). This was due to the use of different reference standards for interpreting clinical categories (CLSI critical concentrations for *Nocardia* spp and other aerobic actinomycetes [23], 2018, for AXO and PK/PD breakpoint of CA-SFM 2025 for FOT [24]). This highlights the major impact of the thresholds defined by different learned societies on the interpretation of MIC and the determination of interpretative clinical categories, highlighting the need to evolve these thresholds as knowledge progresses.

Concerning carbapenems, the MIC data found herein were also consistent with that reported for *N. farcinica*, showing that IMI was better than MERO: 77.4% of S/I isolates versus 22.6%, in the present study; 63% of S/I isolates versus 33% previously published by Brown-Elliott et al. [17]; and 92.3% of S/I isolates versus 84.7% previously published by Yang et al. [18]. Moreover, MERO MIC may be better than those of IMI for certain *Nocardia* species such as *N. wallacei* [17]. However, IMI was also shown slightly better than MERO for *N. cyriacigeorgica* in the present study (93.9% of S/I isolates versus 81.8%), contrary to that previously published by Brown-Elliott et al. [17] and Yang et al. [18] (60% of S/I isolates versus 68%; and 94.45% of S/I isolates versus 97.2%, respectively). In the present study, other species such as *N. gamkensis* and *N. gipuzkoensis* displayed a MIC for MERO 2 dilutions lower than that for IMI. Thus, on a case-by-case basis, MERO could be an alternative for IMI-R *Nocardia* strains, and determining its MIC is necessary in order to prescribe this antibiotic to patients. Although no MERO-specific breakpoint for the genus *Nocardia* is available for interpreting clinical categories, PK/PD breakpoints recommended by the CA-SFM 2025 (S ≤ 2 mg/L, I = 4 and 8 mg/L, *R* ≥ 16 mg/L [24]), used herein, or bacterial interpretative thresholds of the CLSI (S ≤ 4 mg/L, I = 8 mg/L, *R* ≥ 16 mg/L [17, 18]), used by Brown-Elliott et al. and Yang et al., could be used to guide clinicians regarding its potential use. Although these breakpoints differ slightly, the CA-SFM definition of I = S, increased exposure, would allow the use of MERO against *Nocardia* isolates with a MIC up to 8 mg/L.

Concerning TZD, the MIC was lower than that found for LZD, which is consistent with the literature [8]. There are currently no thresholds defined by learned societies (CLSI, EUCAST or CA-SFM) for determining clinical categories for TZD and *Nocardia*. Recently, a study evaluated a PK/PD threshold for TZD and LZD and established it at 0.5 and 1 µg/mL, respectively [30]. Applying these breakpoints in the present study, the frequency of *Nocardia* strains susceptible to TZD appears have a much higher than that susceptible to LZD (93.2% *versus* 29.3%, respectively, data not shown). Furthermore, the PK/PD breakpoint of 1 µg/mL for LZD, which has been adopted by CA-SFM 2025 [24], raises questions about the CLSI’s current breakpoint of 8 µg/mL [23] and suggests that it should be updated. In the present study, one *Nocardia* strain belonging to *N. brasiliensis* exhibited higher MIC to oxazolidinones than the others (TZD 2 µg/mL and LZD 8 µg/mL). It may be worthwhile to sequence this strain to determine whether genetic determinants could explain this reduced susceptibility.

Regarding the comparison of the two AST plates used herein, all FDA criteria (EA, CA, ME, and the upper/lower limit of the 95% CI of the VME rate) were respected for 6 antimicrobials (AUG2, AXO, CIP, CLA, MXF, and TOB) on the 13 antimicrobials contained in both plates. For LZD, the VME rate could not be calculated due to the absence of LZD-resistant *Nocardia* strains, as expected by the recommendations of the CLSI [23]; otherwise, the CA, EA and ME rate criteria were met. The enrichment of the collection with LZD-resistant strains appears to be very difficult, especially when considering the CLSI’s breakpoint.

For 5 antimicrobials (AMI, DOX, IMI, MIN and SXT), the only unmet FDA criterion was the upper limit of 95% CI of VME rate, which exceeded > 7.5%, despite a point estimate and a lower limit of the 95% CI of 0.0%. Achieving an upper limit of the 95% CI < 7.5% for an estimated VME rate of 0.0% would require at least 48 resistant *Nocardia* strains per antimicrobial [25]. For IMI and DOX, this target was nearly reached, and an enrichment with 10 IMI-R strains and 4 DOX-R strains could be considered. However, this appears to be a complicated task for the remaining antimicrobials. For AMI, resistance in *Nocardia* is uncommon, except in the *N. transvalensis* complex [26], which is not commonly involved in human disease [26–29]. In our collection, only 6 *N. wallacei* (belonging to the *N. transvalensis* complex) were identified, including 3 with AMI MIC > 8 mg/L and 2 categorized as S to AMI with an MIC of 8 mg/L. Although this value remains within the S range [23], it is markedly higher than the MIC90 (< 1 mg/L) observed for most other *Nocardia* species. This suggests that AMI resistance in the *N. transvalensis* complex may be difficult be detected by microdilution AST even after 72-hour of incubation. Similarly, SXT resistance was uncommon (4.5%) and consistent with previous studies using microdilution AST technique, reporting resistance rates between 0.9% in a Chinese collection [26] and 6.5% in a Israeli collection [31]. The resistant isolates involved the same species previously reported, namely *N. farcinica*, *N. wallacei* and *N. otitidiscavarium* [26, 31, 32]. For MIN, the Chinese and Israeli studies mentioned above report, as herein, a high proportion of MIN-I strains with MIC of 2–4 µg/mL but very few MIN-R strains (0.9% and 0.0% respectively [26, 31]).

Another point of note is that, for TGC, only the EA was met. The reason for this was that the breakpoint used to define the clinical categories (S ≤ 0.5 µg/mL, *R* > 0.5 µg/mL) also corresponds to the modal MIC of all the strains tested, which divided the overall *Nocardia* population into two almost equal parts between TGC-S (46.6%) and TGC-R (53.4%). As no zone “I” was determined, it is very easy to move from S to R in one MIC dilution, resulting in poor CA, as well as ME and VME rates.

With regard to reproducibility testing, the tests carried out herein do not meet FDA requirements as they were conducted in a single center on only two strains of different species (*S. aureus* and *N. asteroides*). However, for internal use at the LBMR des nocardioses, these tests appear sufficient to ensure reproducible use of the device within the laboratory. 

The main limitations of the present study, other than the lack of resistant strains for certain antimicrobials as mentioned above, were, first, that the FDA criteria could not assessed according to *Nocardia* species stratification because of the limited number of isolates for all species. Second, the reference method used to evaluate the FRNOCAR1 plate was a commercially available ready-to-use plate, RAPMYCOI. Nevertheless, the RAPMYCOI plate is IVD-labeled and has been evaluated and validated by the manufacturer for *Nocardia* spp., its use ensures a certain degree of accuracy of the results, although systematic biases may still be present. Third, certain antimicrobials, such as TZD, MERO, and FOT, were available only on the FRNOCAR1 plate, preventing comparison of the results with a reference method.

Regarding the latter point, the TZD MICs obtained with the FRNOCAR1 for the reference strain *S. aureus* ATCC 29,213 were within the expected target ranges 100% of the time. This result provides at least partial validation of the accuracy and of the reproducibility of the TZD MIC generated by the FRNOCAR1. Although no specific clinical breakpoint has been established for *Nocardia* spp. with TZD, preliminary PK/PD data [30] and reported clinical cases [9–13] suggest that TZD may represent an alternative treatment option for nocardiosis in selected situations such as resistance, adverse effects, or drug-drug interactions. Consequently, MIC values for this antimicrobial could be reported without clinical category interpretation, together with a comment recommending expert consultation prior to its use for the curative treatment of nocardiosis.

For MERO and FOT, the MIC obtained for the reference strain *S. aureus* ATCC 29,213, although reproducible, did not allow conclusions to be drawn regarding the accuracy of the MICs generated by the FRNOCAR1, in the absence of expected MIC ranges established by learned societies (CLSI or CA-SFM). The use of another quality control strain with well-established expected MIC ranges could be considered, such as *Escherichia coli* ATCC 25,922. However, because the expected MIC ranges for this strain are very low—0.03 to 0.125 mg/L for FOT and 0.008 to 0.06 mg/L for MERO—the concentration range available on the FRNOCAR1 would not allow for sufficiently accurate evaluation of these MIC. MERO already appears to be commonly used in certain countries, particularly China and India, especially for the treatment of cerebral nocardiosis [19–21]. In this regard, a recent meta-analysis on cerebral nocardiosis reported that MERO was combined with SXT for the antimicrobial treatment of these infections [33]. In this context, and although additional analyses remain necessary to ensure the accuracy of MERO MIC obtained using the FRNOCAR1, it appears desirable to be able to provide, under specific conditions, a MERO MIC determined by microdilution when available. This could help generate clinical efficacy data when MERO is used for treatment. Consequently, MERO MIC obtained with the FRNOCAR1 could be reported without clinical category interpretation, only upon justified request from an expert clinician, together with a comment indicating that the result is pending further validation for accuracy, interpretative criteria, and clinical correlation.

For FOT, the MICs were very similar to those of AXO, not offering any clear advantage over AXO for the treatment of nocardiosis. Consequently, the FOT MICs obtained with the FRNOCAR1 plate may remain unreported and be used for research purposes only.

In conclusion, for AMI, AUG2, AXO, CLA, CIP, DOX, IMI, LZD, MIN, MXF, SXT, and TOB, the FRNOCAR1 demonstrated good agreement compared with RAPMYCOI for *Nocardia* strains. Furthermore, the FRNOCAR1 plate enables the determination of TZD MIC, a parameter not available with existing ready-to-use commercial plates such as the RAPMYCOI. The LBMR des nocardioses therefore routinely uses the FRNOCAR1 for microdilution AST of *Nocardia* for the following agents: AMI, AUG2, AXO, CLA, CIP, DOX, IMI, LZD, MIN, MXF, SXT, TOB, and TZD. The MERO MIC data generated concurrently could be reported to clinicians upon justified request, accompanied by a comment stating that the results remain provisional pending full validation. The generated FOT MIC data are intended exclusively for research use. This approach allows results to be obtained within a timeframe compatible with clinical management and for antibiotics of therapeutic interest.

## Supplementary Information

Below is the link to the electronic supplementary material.

Supplementary Material 1 (DOCX 471 KB)

## Data Availability

The data presented in this study are available on reasonable request from the corresponding author.
