# Supplementary material for: Nocardia antimicrobial susceptibility testing by microdilution: evaluation of ready-to-use plate including tedizolid and meropenem
Source: Eur J Clin Microbiol Infect Dis. 2026 Jul 4;45(10):3103–15. doi: 10.1007/s10096-026-05588-0 (PMC13676515; doi:10.1007/s10096-026-05588-0)
Supplement: Supplementary file 1 [file 10096_2026_5588_MOESM1_ESM.docx]

Original article – European journal of clinical microbiology and infectious diseases

***Nocardia* antimicrobial susceptibility testing by microdilution: evaluation of ready-to-use plate including tedizolid and meropenem**

Mateo Dutarte^1^, Charlotte Genestet^1^, Gérard Lina^1^, Oana Dumitrescu^1^, Elisabeth Hodille^1*^

^1^ Laboratoire de biologie médicale de référence des nocardioses, Institut des agents infectieux, Laboratoire de biologie médicale multi-site, Groupement Hospitalier Nord, Hospices civils de Lyon, Lyon, France

*corresponding author: [elisabeth.hodille@chu-lyon.fr](mailto:elisabeth.hodille@chu-lyon.fr); Groupement Hospitalier Nord, Hôpital de la Croix Rousse, 121 Boulevard des Canuts, 121 Boulevard des Canuts, 69004 Lyon, France; +33472071111

**Supplementary information**

**Table S1-S15:** Reproducibility results of Sensititre^TM^ FRNOCAR1 plate within “LBMR des nocardioses” by organism: *N. asteroids* ATCC19247, 20 replicates, and *S. aureus* ATCC 29213, 5 replicates; and conformity with minimal inhibitory concentration (MIC) expected value of *Comité de l’Antibiogramme de la Société Française de Microbiologie* (CA-SFM) 2025 for *S. aureus* ATCC 29213 if applicable.

|  | |  | |  | Difference in the number of wells between test result ant test mode | | | | | | | | |
| --- | --- | --- | --- | --- | --- | --- | --- | --- | --- | --- | --- | --- | --- |
| Organism | | Antibiotics | | Test mode  MIC (mg/L) | Off-scale | -2 | -1 | | mode | 1 | | 2 | Off-scale |
|  |  |  |  |  |  |  |  |  | 0 |  |  |  |  |
| *N. asteroides* | | AMI | | ≤0.5 |  |  |  | | 20 |  | |  |  |
| *S. aureus* | |  |  | 2 |  |  |  | | 3 | 2 | |  |  |
| Within-site reproducibility | | | | | 0 | 0 | 0 | | 23 | 2 | | 0 | 0 |
|  |  |  |  |  | The best reproducibility = 25/25, 100.0% | | | | | | | | |
|  |  |  |  |  | The worst reproducibility = 25/25, 100.0% | | | | | | | | |
|  | | | | |  | | | | | | | | |
| *S. aureus* | Antibiotics | | Target MIC (mg/L)^*^ | | Acceptable limit MIC (mg/L)^*^ | | | % MIC in the acceptable limit | | |  | | |
|  | AMI | | 2 | | 1 - 4 | | | 100% | | |  | | |

AMI: amikacin ; * Breakpoint of *Comité de l’Antibiogramme de la Société Française de Microbiologie* (CA-SFM) 2025.

|  | |  | |  | Difference in the number of wells between test result ant test mode | | | | | | | | |
| --- | --- | --- | --- | --- | --- | --- | --- | --- | --- | --- | --- | --- | --- |
| Organism | | Antibiotics | | Test mode | Off-scale | -2 | -1 | | mode | 1 | | 2 | Off-scale |
|  |  |  |  |  |  |  |  |  | 0 |  |  |  |  |
| *N. asteroides* | | AUG2 | | >64 |  |  |  | | 20 |  | |  |  |
| *S. aureus* | |  |  | ≤2/1 |  |  |  | | 5 |  | |  |  |
| Within-site reproducibility | | | | | 0 | 0 | 0 | | 25 | 0 | | 0 | 0 |
|  |  |  |  |  | The best reproducibility = 25/25, 100.0% | | | | | | | | |
|  |  |  |  |  | The worst reproducibility = 25/25, 100.0% | | | | | | | | |
|  | | | | | | | | | | | | | |
| *S. aureus* | Antibiotics | | Target MIC (mg/L)^*^ | | Acceptable limit MIC (mg/L)^*^ | | | % MIC in the acceptable limit | | |  | | |
|  | AUG2 | | 0.25 | | 0.125 - 0.5 | | | Und | | |  | | |

AUG2: amoxicillin-clavulanate; * Breakpoint of *Comité de l’Antibiogramme de la Société Française de Microbiologie* (CA-SFM) 2025; Und: Undetermined because the FRNOCAR1 plate does not allow sufficiently low MIC dilutions for AUG2.

|  |  |  | Difference in the number of wells between test result ant test mode | | | | | | |
| --- | --- | --- | --- | --- | --- | --- | --- | --- | --- |
| Organism | Antibiotics | Test mode | Off-scale | -2 | -1 | mode | 1 | 2 | Off-scale |
|  |  |  |  |  |  | 0 |  |  |  |
| *N. asteroides* | AXO | 64 |  |  | 4 | 14 |  |  | 2 |
| *S. aureus* |  | 4 |  |  |  | 5 |  |  |  |
| Within-site reproducibility | | | 0 | 0 | 4 | 19 | 0 | 0 | 2 |
|  |  |  | The best reproducibility = 25/25, 100.0% | | | | | | |
|  |  |  | The worst reproducibility = 23/25, 92.0% | | | | | | |

AXO: ceftriaxone. None MIC expected value recommended by *Comité de l’Antibiogramme de la Société Française de Microbiologie* (CA-SFM) 2025.

|  | |  | |  | Difference in the number of wells between test result ant test mode | | | | | | | | |
| --- | --- | --- | --- | --- | --- | --- | --- | --- | --- | --- | --- | --- | --- |
| Organism | | Antibiotics | | Test mode | Off-scale | -2 | -1 | | mode | 1 | | 2 | Off-scale |
|  |  |  |  |  |  |  |  |  | 0 |  |  |  |  |
| *N. asteroides* | | CLA | | >8 |  |  |  | | 20 |  | |  |  |
| *S. aureus* | |  |  | ≤ 0.5 |  |  |  | | 5 |  | |  |  |
| Within-site reproducibility | | | | | 0 | 0 | 0 | | 25 | 0 | | 0 | 0 |
|  |  |  |  |  | The best reproducibility = 25/25, 100.0% | | | | | | | | |
|  |  |  |  |  | The worst reproducibility = 25/25, 100.0% | | | | | | | | |
|  | | | | | | | | | | | | | |
| *S. aureus* | Antibiotics | | Target MIC (mg/L)^*^ | | Acceptable limit MIC (mg/L)^*^ | | | % MIC in the acceptable limit | | |  | | |
|  | CLA | | 0.25 | | 0.125-0.5 | | | 100% | | |  | | |

CLA : clarithromycin; * Breakpoint of *Comité de l’Antibiogramme de la Société Française de Microbiologie* (CA-SFM) 2025.

|  | |  | |  | Difference in the number of wells between test result ant test mode | | | | | | | | |
| --- | --- | --- | --- | --- | --- | --- | --- | --- | --- | --- | --- | --- | --- |
| Organism | | Antibiotics | | Test mode | Off-scale | -2 | -1 | | mode | 1 | | 2 | Off-scale |
|  |  |  |  |  |  |  |  |  | 0 |  |  |  |  |
| *N. asteroides* | | CIP | | >16 |  |  |  | | 20 |  | |  |  |
| *S. aureus* | |  |  | ≤ 0.5 |  |  |  | | 5 |  | |  |  |
| Within-site reproducibility | | | | | 0 | 0 | 0 | | 25 | 0 | | 0 | 0 |
|  |  |  |  |  | The best reproducibility = 25/25, 100.0% | | | | | | | | |
|  |  |  |  |  | The worst reproducibility = 25/25, 100.0% | | | | | | | | |
|  | | | | |  | | | | | | | | |
| *S. aureus* | Antibiotics | | Target MIC (mg/L)^*^ | | Acceptable limit MIC (mg/L)^*^ | | | % MIC in the acceptable limit | | |  | | |
|  | CIP | | 0.25 | | 0.125 – 0.5 | | | 100% | | |  | | |

CIP : ciprofloxacin; * Breakpoint of *Comité de l’Antibiogramme de la Société Française de Microbiologie* (CA-SFM) 2025.

|  | |  | |  | Difference in the number of wells between test result ant test mode | | | | | | | | |
| --- | --- | --- | --- | --- | --- | --- | --- | --- | --- | --- | --- | --- | --- |
| Organism | | Antibiotics | | Test mode | Off-scale | -2 | -1 | | mode | 1 | | 2 | Off-scale |
|  |  |  |  |  |  |  |  |  | 0 |  |  |  |  |
| *N. asteroides* | | DOX | | 2 |  |  | 5 | | 14 | 1 | |  |  |
| *S. aureus* | |  |  | ≤0.5 |  |  |  | | 5 |  | |  |  |
| Within-site reproducibility | | | | | 0 | 0 | 5 | | 19 | 1 | | 0 | 0 |
|  |  |  |  |  | The best reproducibility = 25/25, 100.0% | | | | | | | | |
|  |  |  |  |  | The worst reproducibility = 25/25, 100.0% | | | | | | | | |
|  | | | | |  | | | | | | | | |
| *S. aureus* | Antibiotics | | Target MIC (mg/L)^*^ | | Acceptable limit MIC (mg/L)^*^ | | | % MIC in the acceptable limit | | |  | | |
|  | DOX | | 0.25 | | 0.125 – 0.5 | | | 100% | | |  | | |

DOX : doxycycline; * Breakpoint of *Comité de l’Antibiogramme de la Société Française de Microbiologie* (CA-SFM) 2025.

| Organism | Antibiotics | Test mode | Off-scale | -2 | -1 | mode | 1 | 2 | Off-scale |
| --- | --- | --- | --- | --- | --- | --- | --- | --- | --- |
|  |  |  |  |  |  | 0 |  |  |  |
| *N. asteroides* | FOT | >64 |  |  |  | 20 |  |  |  |
| *S. aureus* |  | 2 |  |  |  | 3 | 2 |  |  |
| Within-site reproducibility | | | 0 | 0 | 0 | 23 | 2 | 0 | 0 |
|  |  |  | The best reproducibility = 25/25, 100.0% | | | | | | |
|  |  |  | The worst reproducibility = 25/25, 100.0% | | | | | | |

FOT: cefotaxime. None MIC expected value recommended by *Comité de l’Antibiogramme de la Société Française de Microbiologie* (CA-SFM) 2025.

|  |  |  | Difference in the number of wells between test result ant test mode | | | | | | |
| --- | --- | --- | --- | --- | --- | --- | --- | --- | --- |
| Organism | Antibiotics | Test mode | Off-scale | -2 | -1 | mode | 1 | 2 | Off-scale |
|  |  |  |  |  |  | 0 |  |  |  |
| *N. asteroides* | IMI | 16 |  |  |  | 20 |  |  |  |
| *S. aureus* |  | 1 |  |  |  | 5 |  |  |  |
| Within-site reproducibility | | | 0 | 0 | 0 | 25 | 0 | 0 | 0 |
|  |  |  | The best reproducibility = 25/25, 100.0% | | | | | | |
|  |  |  | The worst reproducibility = 25/25, 100.0% | | | | | | |

IMI: imipenem. None MIC expected value recommended by *Comité de l’Antibiogramme de la Société Française de Microbiologie* (CA-SFM) 2025.

|  | |  | |  | Difference in the number of wells between test result ant test mode | | | | | | | | |
| --- | --- | --- | --- | --- | --- | --- | --- | --- | --- | --- | --- | --- | --- |
| Organism | | Antibiotics | | Test mode | Off-scale | -2 | -1 | | mode | 1 | | 2 | Off-scale |
|  |  |  |  |  |  |  |  |  | 0 |  |  |  |  |
| *N. asteroides* | | LZD | | 4 |  |  | 5 | | 14 | 1 | |  |  |
| *S. aureus* | |  |  | 2 |  |  |  | | 5 |  | |  |  |
| Within-site reproducibility | | | | | 0 | 0 | 5 | | 19 | 1 | | 0 | 0 |
|  |  |  |  |  | The best reproducibility = 25/25, 100.0% | | | | | | | | |
|  |  |  |  |  | The worst reproducibility = 25/25, 100.0% | | | | | | | | |
|  | | | | |  | | | | | | | | |
| *S. aureus* | Antibiotics | | Target MIC (mg/L)^*^ | | Acceptable limit MIC (mg/L)^*^ | | | % MIC in the acceptable limit | | |  | | |
|  | LZD | | 2 | | 1 - 4 | | | 100% | | |  | | |

LZD: linezolid ; * Breakpoint of *Comité de l’Antibiogramme de la Société Française de Microbiologie* (CA-SFM) 2025.

|  |  |  | Difference in the number of wells between test result ant test mode | | | | | | |
| --- | --- | --- | --- | --- | --- | --- | --- | --- | --- |
| Organism | Antibiotics | Test mode | Off-scale | -2 | -1 | mode | 1 | 2 | Off-scale |
|  |  |  |  |  |  | 0 |  |  |  |
| *N. asteroides* | MERO | 2 |  |  |  | 20 |  |  |  |
| *S. aureus* |  | <1 |  |  |  | 5 |  |  |  |
| Within-site reproducibility | | | 0 | 0 | 0 | 25 | 0 | 0 | 0 |
|  |  |  | The best reproducibility = 25/25, 100.0% | | | | | | |
|  |  |  | The worst reproducibility = 25/25, 100.0% | | | | | | |

MERO: meropenem. None MIC expected value recommended by *Comité de l’Antibiogramme de la Société Française de Microbiologie* (CA-SFM) 2025.

|  | |  | |  | Difference in the number of wells between test result ant test mode | | | | | | | | |
| --- | --- | --- | --- | --- | --- | --- | --- | --- | --- | --- | --- | --- | --- |
| Organism | | Antibiotics | | Test mode | Off-scale | -2 | -1 | | mode | 1 | | 2 | Off-scale |
|  |  |  |  |  |  |  |  |  | 0 |  |  |  |  |
| *N. asteroides* | | MIN | | 4 |  |  |  | | 17 | 3 | |  |  |
| *S. aureus* | |  |  | ≤ 0.25 |  |  |  | | 5 |  | |  |  |
| Within-site reproducibility | | | | | 0 | 0 | 0 | | 22 | 3 | | 0 | 0 |
|  |  |  |  |  | The best reproducibility = 25/25, 100.0% | | | | | | | | |
|  |  |  |  |  | The worst reproducibility = 25/25, 100.0% | | | | | | | | |
|  | | | | |  | | | | | | | | |
| *S. aureus* | Antibiotics | | Target MIC (mg/L)^*^ | | Acceptable limit MIC (mg/L)^*^ | | | % MIC in the acceptable limit | | |  | | |
|  | MIN | | 0.125 – 0.25 | | 0.06 – 0.5 | | | 100% | | |  | | |

MIN: minocycline ; * Breakpoint of *Comité de l’Antibiogramme de la Société Française de Microbiologie* (CA-SFM) 2025.

|  | |  | |  | Difference in the number of wells between test result ant test mode | | | | | | | | |
| --- | --- | --- | --- | --- | --- | --- | --- | --- | --- | --- | --- | --- | --- |
| Organism | | Antibiotics | | Test mode | Off-scale | -2 | -1 | | mode | 1 | | 2 | Off-scale |
|  |  |  |  |  |  |  |  |  | 0 |  |  |  |  |
| *N. asteroides* | | MXF | | 2 |  |  | 1 | | 17 | 2 | |  |  |
| *S. aureus* | |  |  | ≤ 0.5 |  |  |  | | 5 |  | |  |  |
| Within-site reproducibility | | | | | 0 | 0 | 1 | | 22 | 2 | | 0 | 0 |
|  |  |  |  |  | The best reproducibility = 25/25, 100.0% | | | | | | | | |
|  |  |  |  |  | The worst reproducibility = 25/25, 100.0% | | | | | | | | |
|  | | | | |  | | | | | | | | |
| *S. aureus* | Antibiotics | | Target MIC (mg/L)^*^ | | Acceptable limit MIC (mg/L)^*^ | | | % MIC in the acceptable limit | | |  | | |
|  | MXF | | 0.03 – 0.06 | | 0.016 – 0.125 | | | Und | | |  | | |

MXF: moxifloxacine; * Breakpoint of *Comité de l’Antibiogramme de la Société Française de Microbiologie* (CA-SFM) 2025; Und: Undetermined because the FRNOCAR1 plate does not allow sufficiently low MIC dilutions for MXF.

|  | |  | |  | Difference in the number of wells between test result ant test mode | | | | | | | | |
| --- | --- | --- | --- | --- | --- | --- | --- | --- | --- | --- | --- | --- | --- |
| Organism | | Antibiotics | | Test mode | Off-scale | -2 | -1 | | mode | 1 | | 2 | Off-scale |
|  |  |  |  |  |  |  |  |  | 0 |  |  |  |  |
| *N. asteroides* | | SXT | | 0.5/9.5 |  |  |  | | 20 |  | |  |  |
| *S. aureus* | |  |  | ≤ 0.1/2.38 |  |  |  | | 5 |  | |  |  |
| Within-site reproducibility | | | | | 0 | 0 | 0 | | 25 | 0 | | 0 | 0 |
|  |  |  |  |  | The best reproducibility = 25/25, 100.0% | | | | | | | | |
|  |  |  |  |  | The worst reproducibility = 25/25, 100.0% | | | | | | | | |
|  | | | | |  | | | | | | | | |
| *S. aureus* | Antibiotics | | Target MIC (mg/L)^*^ | | Acceptable limit MIC (mg/L)^*^ | | | % MIC in the acceptable limit | | |  | | |
|  | SXT | | ≤ 0.5/9.5 | | / | | | 100% | | |  | | |

SXT: trimethoprim-sulfamethoxazole ; * Breakpoint of *Comité de l’Antibiogramme de la Société Française de Microbiologie* (CA-SFM) 2025.

|  | |  | |  | Difference in the number of wells between test result ant test mode | | | | | | | | |
| --- | --- | --- | --- | --- | --- | --- | --- | --- | --- | --- | --- | --- | --- |
| Organism | | Antibiotics | | Test mode | Off-scale | -2 | -1 | | mode | 1 | | 2 | Off-scale |
|  |  |  |  |  |  |  |  |  | 0 |  |  |  |  |
| *N. asteroides* | | TOB | | ≤1 |  |  |  | | 20 |  | |  |  |
| *S. aureus* | |  |  | ≤1 |  |  |  | | 5 |  | |  |  |
| Within-site reproducibility | | | | | 0 | 0 | 0 | | 25 | 0 | | 0 | 0 |
|  |  |  |  |  | The best reproducibility = 25/25, 100.0% | | | | | | | | |
|  |  |  |  |  | The worst reproducibility = 25/25, 100.0% | | | | | | | | |
|  | | | | |  | | | | | | | | |
| *S. aureus* | Antibiotics | | Target MIC (mg/L)^*^ | | Acceptable limit MIC (mg/L)^*^ | | | % MIC in the acceptable limit | | |  | | |
|  | TOB | | 0.25 – 0.5 | | 0.125 - 1 | | | 100% | | |  | | |

TOB: tobramycin; * Breakpoint of *Comité de l’Antibiogramme de la Société Française de Microbiologie* (CA-SFM) 2025.

|  | |  | |  | Difference in the number of wells between test result ant test mode | | | | | | | | |
| --- | --- | --- | --- | --- | --- | --- | --- | --- | --- | --- | --- | --- | --- |
| Organism | | Antibiotics | | Test mode | Off-scale | -2 | -1 | | mode | 1 | | 2 | Off-scale |
|  |  |  |  |  |  |  |  |  | 0 |  |  |  |  |
| *N. asteroides* | | TZD | | 0,5 |  |  |  | | 13 | 7 | |  |  |
| *S. aureus* | |  |  | 0,25 |  |  |  | | 5 |  | |  |  |
| Within-site reproducibility | | | | | 0 | 0 | 0 | | 18 | 7 | | 0 | 0 |
|  |  |  |  |  | The best reproducibility = 25/25, 100.0% | | | | | | | | |
|  |  |  |  |  | The worst reproducibility = 25/25, 100.0% | | | | | | | | |
|  | | | | |  | | | | | | | | |
| *S. aureus* | Antibiotics | | Target MIC (mg/L)^*^ | | Acceptable limit MIC (mg/L)^*^ | | | % MIC in the acceptable limit | | |  | | |
|  | TZD | | 0.25 – 0.5 | | 0.25 - 1 | | | 100% | | |  | | |

TZD: tedizolid; * Breakpoint of *Comité de l’Antibiogramme de la Société Française de Microbiologie* (CA-SFM) 2025.

A

B

**Figure S1** Plates layout for Sensititre^TM^ FRNOCAR1 (A) and Sensititre^TM^ Myco RAPMYCOI (B). AMI: amikacin; AXO: ceftriaxone; AUG2: amoxicillin-clavulanate; CIP: ciprofloxacin; CLA: clarithromycin; DOX: doxycycline; FEP: cefepime; FOT: cefotaxime; FOX: cefoxitine; IMI: imipenem; LZD: linezolid; MERO: meropenem; MIN: minocycline; MXF: moxifloxacin; SXT: trimethoprim-sulfamethoxazole; TGC: tigecycline; TOB: tobramycin TZD: tedizolid

B

A

D

C

F

E

G

H

J

I

L

K

N

M

*N. cyriacigeorgica*

*N. nova* complex

*N. farcinica*

Other *Nocardia* species

**Figure S2** *Nocardia* (n=133) MIC distribution of ciprofloxacin (CIP, panels A and B), clarithromycin (CLA, panel C and D), doxycycline (DOX, panels E and F), minocycline (MIN, panels G and H), moxifloxacin (MXF, panels I and J), tigecycline (TGC, panels K and L), and tobramycin (TOB, panels M and N) according to species. MIC derived from data originating from RAPMYCOI plate for the panels A, C, E, G, I, and K and from FRNOCAR1 plate for the panels B, D, F, H, J, L, and N. S: Susceptible; I: Intermediate; R: Resistant. Dotted lines: critical concentrations of CLSI for *Nocardia* spp and other aerobic actinomycetes, except for TGC: PK/PD breakpoint of *Comité de l’Antibiogramme de la Société Française de Microbiologie* (CA-SFM) 2025.

B

A

*N. cyriacigeorgica*

*N. nova* complex

*N. farcinica*

Other *Nocardia* species

**Figure S3** *Nocardia* (n=133) MIC distribution of cefepime (FEP, panel A), and cefoxitin (FOX, panel B), according to species. MIC derived from data originating from RAPMYCOI plate. S: Susceptible; I: Intermediate; R: Resistant. Dotted lines: critical concentrations of CLSI for *Nocardia* spp and other aerobic actinomycetes for FOX; and PK/PD breakpoint of *Comité de l’Antibiogramme de la Société Française de Microbiologie* (CA-SFM) 2025 for FEP.

*N. cyriacigeorgica*

*N. nova* complex

*N. farcinica*

Other *Nocardia* species

**Figure S4** *Nocardia* (n=133) MIC distribution of cefotaxime (FOT), according to species. MIC derived from data originating from FRNOCAR1 plate. S: Susceptible; I: Intermediate; R: Resistant. Dotted lines: PK/PD breakpoint of *Comité de l’Antibiogramme de la Société Française de Microbiologie* (CA-SFM) 2025.
